# Skeletal Ryanodine Receptors Are Involved in Impaired Myogenic Differentiation in Duchenne Muscular Dystrophy Patients

**DOI:** 10.3390/ijms222312985

**Published:** 2021-11-30

**Authors:** Pierre Meyer, Cécile Notarnicola, Albano C. Meli, Stefan Matecki, Gérald Hugon, Jérémy Salvador, Mirna Khalil, Léonard Féasson, Claude Cances, Jérôme Cottalorda, Isabelle Desguerre, Jean-Marie Cuisset, Pascal Sabouraud, Alain Lacampagne, Hugues Chevassus, François Rivier, Gilles Carnac

**Affiliations:** 1 PhyMedExp, University of Montpellier, Inserm, CNRS, 34295 Montpellier, France; cecile.notarnicola@inserm.fr (C.N.); albano.meli@inserm.fr (A.C.M.); stephan.matecki@umontpellier.fr (S.M.); gerald.hugon@inserm.fr (G.H.); jeremy.salvador@inserm.fr (J.S.); alain.lacampagne@inserm.fr (A.L.); f-rivier@chu-montpellier.fr (F.R.); gilles.carnac@inserm.fr (G.C.); 2 Reference Centre for Neuromuscular Diseases AOC, Clinical Investigation Centre, Pediatric Neurology Department, Montpellier University Hospital, 34000 Montpellier, France; 3 Clinical Investigation Center, Montpellier University Hospital, 34000 Montpellier, France; mirna-khalil@chu-montpellier.fr (M.K.); h-chevassus@chu-montpellier.fr (H.C.); 4 Myology Unit, Reference Center for Neuromuscular Diseases Euro-NmD, Inter-University Laboratory of Human Movement Sciences—EA7424, University Hospital of Saint-Etienne, 42055 Saint-Etienne, France; leonard.feasson@univ-st-etienne.fr; 5 Reference Center for Neuromuscular Diseases AOC, Pediatric Neurology Department, Toulouse University Hospital, 3100 Toulouse, France; cances.c@chu-toulouse.fr; 6 Pediatric Clinical Research Unit, Pediatric Multi-thematic Module CIC 1436, Toulouse Children’s Hospital, 31300 Toulouse, France; 7 Pediatric Orthopedic and Plastic Surgery Department, Montpellier University Hospital, 34295 Montpellier, France; j-cottalorda@chu-montpellier.fr; 8 Reference Center for Neuromuscular Diseases Paris Nord-Ile-de-France-Est, Pediatric Neurology Department, Necker Enfant Malades University Hospital, Assistance Publique des Hôpitaux de Paris Centre, Paris University, 75019 Paris, France; isabelle.desguerre@aphp.fr; 9 Reference Center for Neuromuscular Diseases Nord-Ile-de-France-Est, Pediatric Neurology Department, Lille University Hospital, 59000 Lille, France; jm.cuisset@free.fr; 10 Reference Center for Neuromuscular Diseases Nord-Ile-de-France-Est, Pediatric Neurology Department, Reims University Hospital, 51100 Reims, France; psabouraud@chu-reims.fr

**Keywords:** Duchenne muscular dystrophy, ryanodine receptor, myogenesis, endomysial fibrosis, human

## Abstract

Duchenne muscular dystrophy (DMD) is characterized by progressive muscle wasting following repeated muscle damage and inadequate regeneration. Impaired myogenesis and differentiation play a major role in DMD as well as intracellular calcium (Ca^2+^) mishandling. Ca^2+^ release from the sarcoplasmic reticulum is mostly mediated by the type 1 ryanodine receptor (RYR1) that is required for skeletal muscle differentiation in animals. The study objective was to determine whether altered RYR1-mediated Ca^2+^ release contributes to myogenic differentiation impairment in DMD patients. The comparison of primary cultured myoblasts from six boys with DMD and five healthy controls highlighted delayed myoblast differentiation in DMD. Silencing *RYR1* expression using specific si-RNA in a healthy control induced a similar delayed differentiation. In DMD myotubes, resting intracellular Ca^2+^ concentration was increased, but RYR1-mediated Ca^2+^ release was not changed compared with control myotubes. Incubation with the RYR-calstabin interaction stabilizer S107 decreased resting Ca^2+^ concentration in DMD myotubes to control values and improved calstabin1 binding to the RYR1 complex. S107 also improved myogenic differentiation in DMD. Furthermore, intracellular Ca^2+^ concentration was correlated with endomysial fibrosis, which is the only myopathologic parameter associated with poor motor outcome in patients with DMD. This suggested a potential relationship between RYR1 dysfunction and motor impairment. Our study highlights RYR1-mediated Ca^2+^ leakage in human DMD myotubes and its key role in myogenic differentiation impairment. RYR1 stabilization may be an interesting adjunctive therapeutic strategy in DMD.

## 1. Introduction

Duchenne muscular dystrophy (DMD) is a X-recessive disease that affects about 1 in 3500 male births [1]. It is caused by a lack of the sarcolemmal protein dystrophin and is clinically characterized by progressive skeletal muscle degeneration and regeneration, resulting in muscle weakness by the exhaustion of regenerative capacity [2]. Although the dystrophin gene was discovered by Monaco et al. more than 30 years ago, a curative therapy has not been found yet [3,4].

Dystrophin is a 427 kDa structural protein that is localized in the inner part of the sarcolemma and maintains muscle fiber integrity by linking intracellular cytoskeletal actin to the extracellular matrix through its association with a group of plasma membrane glycoproteins, known as the dystrophin–glycoprotein complex [5,6]. Due to dystrophin, which plays an essential role in sarcolemma stability, dystrophin-deficient muscle fibers are prone to chronic membrane damage [7]. However, DMD onset and progression are not fully explained by this sarcolemma fragility, and the exact cause of muscle fiber death is still unclear. The most widely accepted pathological mechanism includes membrane fragility and increased resting intracellular Ca^2+^ levels that induces calpain activation, protein degradation, mitochondrial Ca^2+^ release and, ultimately, fiber necrosis [8,9,10,11,12,13]. Many studies have reported elevated intracellular Ca^2+^ levels in dystrophic muscles from mdx mice (a DMD mouse model) and in patients with DMD compared with normal muscles [10,12,13,14,15,16,17]. Moreover, a transcriptomic analysis of DMD muscle samples identified alterations or upregulation of genes involved in calcium handling [18]. The mechanism of this intracellular Ca^2+^ mishandling is complex and involves trans-sarcolemmal Ca^2+^ fluxes, sarcoplasmic reticulum (SR) Ca^2+^ leakage and abnormal SR Ca^2+^ levels [8,19].

The crosstalk between muscle excitation–contraction and Ca^2+^ dynamics has been extensively studied, but little is known about its role in muscle formation, growth and regeneration. During skeletal muscle development, muscle progenitor cells become mature muscle fibers after the fusion of multinucleated myotubes and then myofibers [20]. The transcription factors PAX3 and PAX7 are expressed by progenitor cells, but they are progressively downregulated during differentiation concomitantly with the induction of myogenic markers (e.g., MYF5 and MYOD) and differentiation markers (e.g., myogenin) [20,21]. At a more advanced stage of differentiation, these cells express muscle-specific proteins, such as actin alpha 1 (ACTA1) and creatine kinase M-type (CKM). In addition, a subset of myogenic stem cells, called muscle satellite cells, remains quiescent in the maturing and adult skeletal muscle and plays a central role in muscle regeneration. After muscle injury, muscle satellite cells are activated and proceed towards the myogenic program in order to repair and replenish the injured tissue [21,22,23]. Ca^2+^ signaling during skeletal myogenesis (muscle development, growth and regeneration) has been studied in various species, from invertebrates to mammals, and a wide range of proteins and signaling partners have been implicated [24]. For instance, calcineurin and calmodulin kinase, two Ca^2+^-dependent enzymes [25,26,27], are needed for the activation of myogenin and MEF2, which are two muscle-specific transcription factors that are expressed early during differentiation. Moreover, an increase in resting Ca^2+^ is required during myoblasts fusion [28]. In skeletal muscle, dynamic Ca^2+^ release from SR is mostly mediated by type 1 ryanodine receptors (RYR1). RYR1 is essential for muscle contraction through its physical coupling with calstabin1 (calcium channel stabilizing binding protein, also known as FKBP12) [29]. Moreover, some studies demonstrated that RYR1 is required during mouse and *Xenopus laevis* skeletal myogenesis and differentiation [30,31].

Many studies in vitro and also in animal (mdx mice) models and human muscle samples indicate that, in DMD, impaired or delayed muscle cell differentiation is one of the leading causes of disease progression [32,33,34,35,36,37]. Moreover, in mdx skeletal muscles, RYR1 S-nitrolysation increased, resulting in calstabin1 depletion in the channel complex and resulting in leaky RYR1 channels [38]. Leaky RYR1 channels could be implicated in various muscular dystrophy forms linked to mutations in genes encoding components of the dystrophin-glycoprotein complex in mice [39]. The calstabin1-RYR1 association can be preserved by stabilizing compounds called Rycals, such as S107 that stabilizes the interaction between calstabin1 and S-nitrosylated RYR channels, favors their closed conformation eventualy inhibits SR Ca^2+^ leaks [40,41,42]. In young mdx mice treated with S107 for 2 weeks, the biochemical and histologic signs of muscle damage are reduced and muscle function and exercise performance are improved [38]. However, little is known about RYR1 function in human DMD skeletal muscle differentiation. Indeed, mouse models are useful for understanding disease mechanisms and for preclinical investigations, but the obtained data are often difficult to translate to humans, especially due to discrepancies in size, pathophysiology and clinical outcome criteria [43]. We hypothesized that RYR1-mediated Ca^2+^ mishandling could be responsible for impaired myogenic differentiation in human DMD. Therefore, here, we performed ex vivo analyses to compare differentiation and RYR1 function in primary cultured myotubes isolated from biopsies of boys with DMD and healthy controls. We found that myotube differentiation is delayed in DMD compared to healthy control and that RYR1 is required during this process. We then observed a RYR1-mediated Ca^2+^ leakage secondary to reduced RYR1 interaction with calstabin1 in DMD. Rycal compound S107 prevented calstabin1 depletion and improved DMD myogenic differentiation. Furthermore, the abnormal intracellular Ca^2+^ levels were correlated with endomysial fibrosis, the only known DMD feature correlated with poor motor outcome [44]. Altogether, our study emphasizes the key role of RYR1-mediated intracellular Ca^2+^ mishandling in myogenic differentiation (and to some extent in the motor outcome) in DMD and the potential value of RYR stabilizers as adjunctive therapy in human dystrophinopathies.

## 2. Results

### 2.1. Myoblast Differentiation Is Delayed in DMD Patients

**Figure 1:**
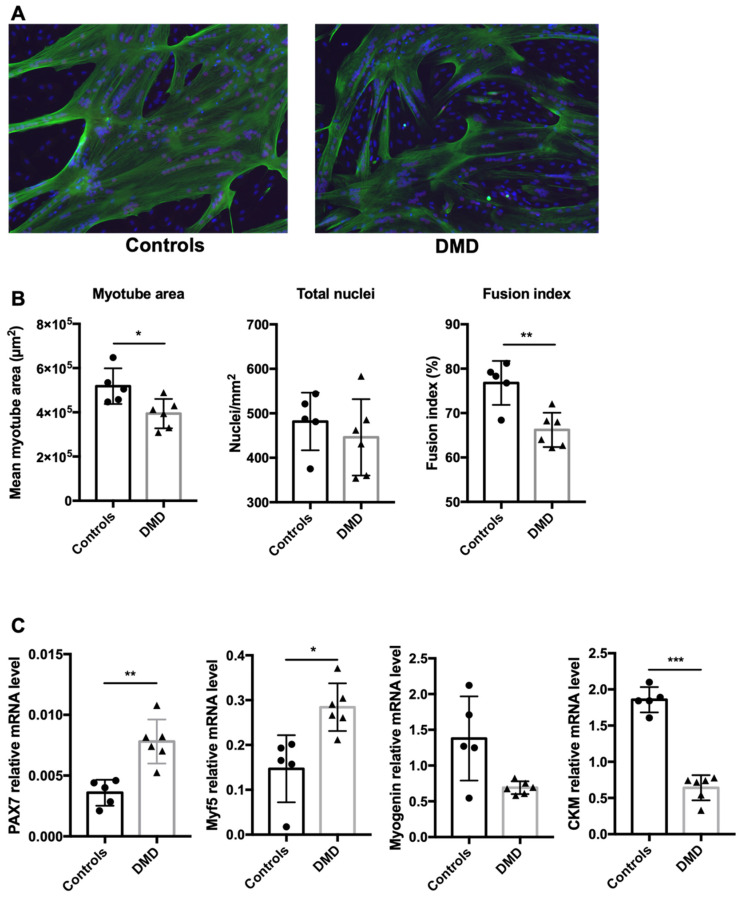
Myoblast differentiation is delayed in DMD patients. (**A**) Representative examples of immunofluorescence analysis of differentiated myotubes from healthy controls (*n* = 5) and patients with DMD (*n* = 6). At confluence, myoblasts were switched to differentiation medium for 3–4 days. Then, cells were stained with an anti-troponin T antibody (green) and DAPI (blue) to determine myotube morphology based on three parameters (**B**): mean myotube area (Troponin T expressing cells with at least 3 merged nuclei), total number of nuclei and fusion index (nuclei per myotube/total nuclei). (**C**) Gene expression analysis by quantitative RT-PCR to monitor muscle cell differentiation in myotubes from healthy controls and patients with DMD. * *p* < 0.05, ** *p* < 0.01, *** *p* < 0.005.

Troponin T immunostaining (a cytoskeletal protein expressed exclusively in myotubes) and nuclei labeling showed a similar mean number of nuclei in cultured myotubes from healthy boys (controls, *n* = 5) and boys with DMD (*n* = 6) (Figure 1A) (481.7 ± 37 nuclei/mm^2^ and 445.8 ± 55 nuclei/mm^2^, respectively, *p* = 0.6), but a reduction in the myotube area (Troponin T expressing cells; 0.518 ± 0.036 mm^2^ and 0.394 ± 0.033 mm^2^, *p* = 0.035) and fusion index (nuclei per myotube/total nuclei; 76.8 ± 2.9% and 66.2 ± 2.3%, *p* = 0.005) in DMD samples (Figure 1B). To further analyze the muscle differentiation stages, we characterized the expression of genes implicated in this process by reverse transcription quantitative PCR (RT-qPCR) (Figure 1C) and found that the expression of *PAX7* (controls 0.0031 ± 0.001 and DMD 0.0078 ± 0.001, *p* = 0.013) and *MYF5* (controls 0.175 ± 0.014 and DMD 0.285 ± 0.033, *p* = 0.043), two transcription factors expressed at immature stages, increased, and the differentiation markers *CKM* (controls 1.85 ± 0.14 and DMD 0.64 ± 0.1, *p* = 0.001) and myogenin (controls 1.305 ± 0.456 and DMD 0.694 ± 0.070, *p* = 0.2) decreased in DMD samples compared with controls, although the latter was not significant [20,21]. This indicated that myogenic differentiation was delayed in DMD samples.

### 2.2. RYR1 Plays a Key Role during Early Human Myoblast Differentiation

As an increase in resting Ca^2+^ is needed during myoblast fusion [28] and RYR1 is required during skeletal muscle differentiation in animal models [30,31], we hypothesized that this channel could play a role in human myoblast differentiation.

**Figure 2:**
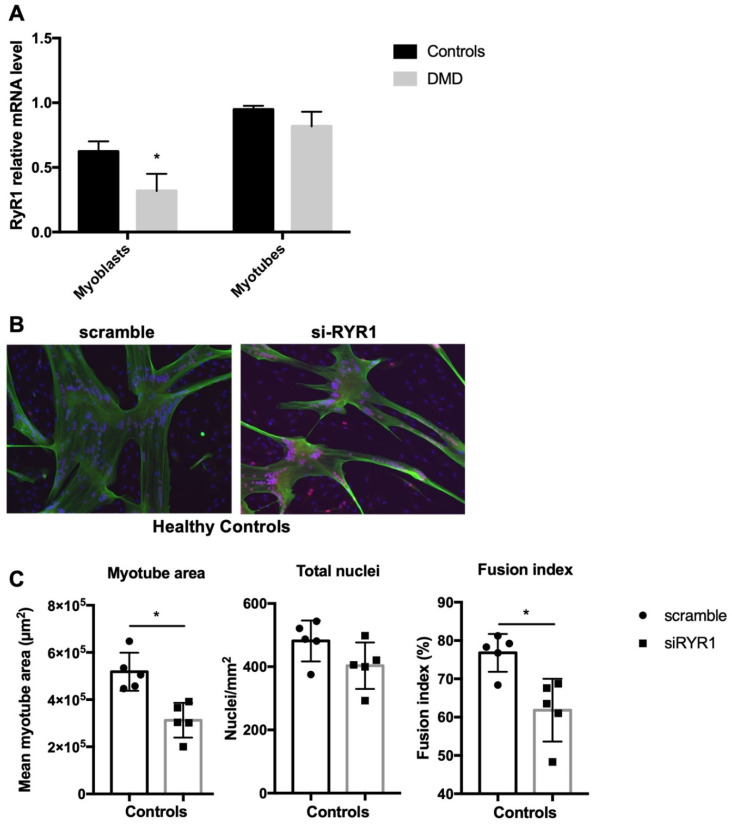
RYR1 plays a key role during early human myoblast differentiation. (**A**) *RYR1* mRNA expression levels were assessed in control (*n* = 5) and DMD (*n* = 6) myoblasts at confluence and in myotubes at day 3–4 of differentiation by quantitative RT-PCR. (**B**) Representative immunofluorescence images of human control myotubes in which *RYR1* was silenced or not (scramble). Myoblasts at 80% of confluence (*n* = 5) were incubated with *RYR1*-specific or scramble siRNAs and then grown in differentiation medium for 3–4 days. Myotubes were stained for troponin T (green) and DAPI (blue) to determine myotube morphology using three parameters (**C**): total myotube area (cells with at least 3 merged nuclei), total number of nuclei and fusion index (nuclei per myotube/total nuclei). * *p* < 0.05.

We first investigated *RYR1* mRNA expression levels in the control (*n* = 5) and DMD (*n* = 6) myoblasts at 80% of confluence and in myotubes after incubation in differentiation medium for 3 to 4 days. *RYR1* mRNA expression was significantly downregulated in DMD myoblasts (controls 0.623 ± 0.079 and DMD 0.318 ± 0.132, *p* = 0.04) but not in differentiated myotubes (controls 0.948 ± 0.028 and DMD 0.818 ± 0.112, *p* = 0.31) (Figure 2A). In skeletal muscle extracts, *RYR1* mRNA expression levels were significantly lower in DMD than control muscle samples. However, this difference was lost when data were normalized to *ACTA1* expression levels, a muscle differentiation marker, to take into account the loss of skeletal tissue secondary to fibrosis and impaired regeneration in DMD (Supplementary Materials, Figure S1).

In order to determine whether RYR1 is required for human myoblast differentiation, we transfected confluent control and DMD myoblasts with a *RYR1*-specific siRNA (si*RYR1*) or with scramble siRNA. In both controls and DMD myotubes, the *RYR1* mRNA level decreased by 63–67% in cultures transfected with the *RYR1* siRNA compared with scramble siRNA. In addition, si*RYR1* efficiently decreased RYR1 protein levels (Supplementary Materials, Figure S2). Even if we cannot explain how the partial reduction in *RYR1* mRNA causes a complete absence of the RYR1 protein detection, we cannot rule out the possibility of translational regulation that could differentially modulate the amount of RYR1 protein. Immunostaining control myoblasts transfected with si*RYR1* or scramble siRNA did not reveal any differences in the total number of nuclei between conditions, but *RYR1* silencing induced a decrease in myotube area (−39.8%, *p* = 0.03) and fusion index (−19.8%, *p* = 0.03) (Figure 2B,C). In DMD cells, *RYR1* silencing affected myoblast differentiation (Supplementary Figure S3), suggesting that RYR1 is required for myogenesis also in DMD myoblasts. Altogether, these data indicate that *RYR1* mRNA expression level changes during the early stages of myogenesis and that RYR1 is required for myogenic differentiation in healthy and also dystrophic skeletal muscle.

### 2.3. RYR1-Mediated Calcium Homeostasis Is Altered in DMD Myotubes

**Figure 3:**
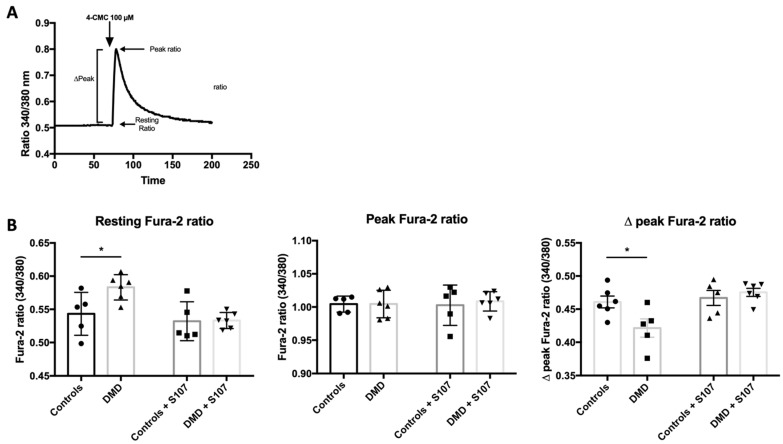
Human DMD myotubes exhibit resting RYR1-mediated calcium leakage that is improved by the RYR stabilizer S107. (**A**) Experimental design: in cultured myotubes loaded with Fura-2, intracellular Ca^2+^ changes were induced by an addition of 4-CMC and monitored by recording the Fura-2 signal emission at 340 and 380 nm. The signal intensities at 340 and 380 nm were used to calculate the signal 340/380 ratio at rest, the peak fluorescence ratio and the ∆Peak. (**B**) Quantification of the resting, peak and ∆Peak Fura-2 (340/380) ratio values in untreated and S107-treated myotubes from healthy controls (*n* = 5) and patients with DMD (*n* = 6). * *p* < 0.05.

To confirm that RYR1 is functional in DMD myotubes, after Fura-2 loading (a dye to measure intracellular Ca^2+^), we stimulated myotubes with 100 µM 4-chloro-*meta*-cresol (4-CmC) in Ca^2+^-free medium to induce RYR1-mediated SR Ca^2+^ release. 4-CmC is a non-physiological RYR-stimulating agent that induces Ca^2+^ release in muscle tissue [45,46,47] (Figure 3A). In order to prevent inositol 1,4,5-trisphosphate receptor (IP3R)-mediated SR Ca^2+^ release, we stimulated cells in the presence of 10 µM xestospongin C, a membrane-permeable specific IP3R inhibitor [48]. Quantification of intracellular free Ca^2+^ by recording Fura-2 fluorescence in myotubes showed that, at rest, the ratio of the Fura-2 signals emitted at 340 and 380 nm (Fura-2 ratio) was higher in DMD than control myotubes (0.583 ± 0.008 and 0.543 ± 0.013, *p* = 0.02). However, after the addition of 4-CmC, the peak Fura-2 ratio values were similar in both groups (1.005 ± 0.008 and 1.003 ± 0.005, *p* = 0.46), whereas the ∆peak Fura-2 ratio was lower in DMD myotubes (0.461 ± 0.009 and 0.421 ± 011, *p* = 0.02) (Figure 3B). The absence of Ca^2+^ release after stimulation with 4-CmC in *RYR1*-silenced myotubes confirmed 4-CmC-specificity for RYR1-dependent Ca^2+^ release. These results show that, in DMD myotubes, the RYR1 opening is normal after stimulation, but intracellular Ca^2+^ concentration increased at rest.

### 2.4. Calcium Leakage Is Improved by RYR1 Stabilizer S107 in DMD Myotubes

The Rycal S107, a 1,4–benzothiazepine derivative, inhibits calstabin1 depletion from RYR1 channels and improves muscle strength in mdx mice and also in 24-month-old mice with age-related muscle weakness and in ß-sarcoglycan-deficient (Sgcb^−/−^) mice, a model of limb-girdle muscular dystrophy [38,39,40].

In order to assess RYR1 implication in the dystrophic myotubes elevated Ca^2+^ intracellular concentration, we examined whether RYR-stabilizer S107 could reverse these phenomena by adding S107 (concentration 10 µM) to DMD myoblasts reaching 80% confluence and analyzing differentiated myotubes at day 4. We found a normalization of Fura-2 ratio during the resting state (resting Fura-2 (340/380) ratio DMD 0.533 ± 0.005 and controls 0.540 ± 0.014, *p* = 0.64) and after 4-CmC stimulation (peak Fura-2 (340/380) ratio DMD 1.003 ± 0.11 and controls 1.009 ± 0.006, *p* = 0.69) (Figure 3B). In accordance with the results obtained in mdx mice [38], calstabin1 co-immunoprecipitation with RYR1 was reduced in untreated but not in S107-treated DMD samples compared with control samples (Supplementary Figure S4). These results confirmed that leaky RYR1s are responsible for increased cytosolic Ca^2+^ levels in human dystrophic myotubes and that improvement of calstabin1 binding to RYR1 by Rycal stabilizer S107 corrects this Ca^2+^ mishandling.

### 2.5. Inhibiting RYR1 Ca^2+^ Leak Improves Myogenic Differentiation in DMD Myoblasts

**Figure 4:**
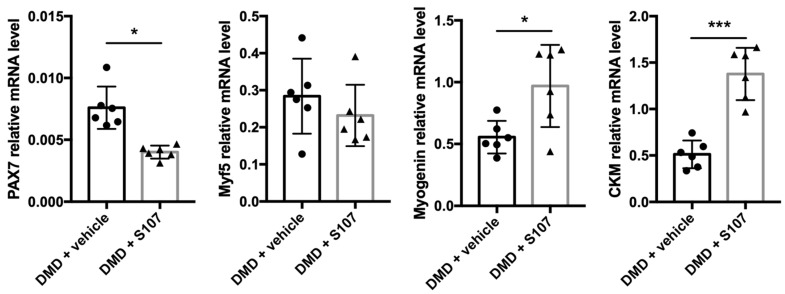
The RYR stabilizer S107 improves myoblast differentiation in DMD. Gene expression analysis by quantitative RT-PCR in myotubes from patients with DMD (*n* = 6) by quantitative RT-PCR. Vehicle or S107 was added to confluent myoblasts before differentiation and myotubes were analyzed after 3 to 4 days of differentiation. * *p* < 0.05, *** *p* < 0.001.

In order to thoroughly investigate S107 effects, we monitored the expression of regulatory myogenic factors by RT-qPCR in myotubes obtained by differentiating myoblasts in the presence or not of 10 µM of S107. The expression of the early myogenic regulatory markers Pax7 (controls 0.0076 ± 0.0011 and S107 0.004 ± 0.0003, *p* = 0.02) and to a lower extent (not significant) Myf5 (controls 0.284 ± 0.066 and S107 0.232 ± 0.053, *p* = 0.5) was reduced in S107-treated compared with untreated DMD cells. Conversely, the expression of terminal differentiation markers, such as myogenin (controls 0.555 ± 0.054 and S107 0.969 ± 0.136, *p* = 0.02) and CKM (controls 0.513 ± 0.955 and S107 1.387 ± 0.155, *p* = 0.003), increased (Figure 4). These data support the hypothesis that delayed myogenic differentiation in DMD skeletal muscle cells could be improved by stabilizing calstabin1 binding to RYR1 to prevent Ca^2+^ leakage.

### 2.6. Endomysial Fibrosis Is Correlated with Elevated Intracellular Ca^2+^ Concentration

**Figure 5:**
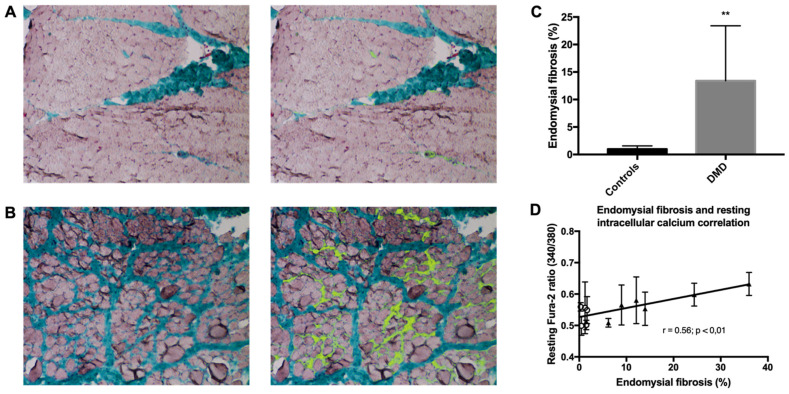
Endomysial fibrosis is correlated with elevated resting intracellular calcium concentration. (**A**,**B**) Representative images showing color segmentation of muscle sections stained with Masson trichrome (left) and with pseudo-color (yellow) to highlight endomysial fibrosis (right) in one healthy control muscle biopsy (**A**) with minimal (1.7%) endomysial fibrosis (biopsy of a 10-year-old boy) and one DMD muscle biopsy (**B**) with advanced (24%) endomysial fibrosis (biopsy of a 5-year-old boy). (**C**) Quantification of endomysial fibrosis in muscle biopsies of healthy controls (*n* = 5) and patients with DMD (*n* = 6). ** *p* < 0.01. (**D**) Correlation between the endomysial fibrosis rate and the Fura-2 ratio at rest in patients with DMD (*n* = 6, triangles) and healthy controls (*n* = 5, circles) (Spearman’s rank correlation coefficient r = 0.56, *p* < 0.01).

Histological analysis of the muscle biopsies from boys with DMD (*n* = 6) and healthy controls (*n* = 5) showed that the endomysial fibrosis rate (Masson’s trichrome staining) was higher in DMD samples than controls (1.5 to 36.8% vs. 0.3 to 1.7%; median values: 12.5% vs. 0.9%, *p* < 0.01) (Figure 5A–C). In order to assess whether elevated intracellular Ca^2+^ could be a prognostic factor in patients with DMD, we compared the Fura-2 ratio at rest and the endomysial fibrosis rate, which is a previously described marker of motor prognosis in DMD [44], and found a significant correlation (Spearman’s correlation, *r* = 0.56, *p* = 0.0016) (Figure 5D). This suggests a potential involvement of RYR1-dependent SR Ca^2+^ leakage and of Ca^2+^ homeostasis mishandling in DMD severity.

## 3. Discussion

In this study, we investigated RYR1’s role in the impaired differentiation of dystrophin-deficient primary human skeletal myogenic cells. We found that myoblast differentiation was delayed also in primary myoblasts from healthy controls in which *RYR1* was silenced. Moreover, in resting DMD myotubes, we observed a RYR1-dependent calcium leakage that could be reversed by stabilizing calstabin1 binding to RYR1 with the Rycal S107. S107 also improved DMD myoblast differentiation.

It should be noted that we evaluated gene expression in cultured primary muscle cells isolated from biopsy samples obtained at diagnosis when all boys were still untreated. Furthermore, our culture system allowed us to study myogenesis and calcium homeostasis only in dystrophin-lacking muscle cells without taking into account the extracellular environment interference (e.g., mechanical load and/or inflammation). Many studies, particularly gene expression profiling analyses, were based on RNA extracts from crushed muscle biopsy samples [18,49], thus making it difficult to distinguish between genes specifically expressed in muscle cells and in their environment (e.g., fibroblasts and macrophages).

We found that DMD skeletal muscle cell differentiation was delayed and hypothesized that RYR1 may play a key role in this phenotype. To our knowledge, RYR1 implication in skeletal muscle differentiation has never been studied in DMD, although this receptor is a major source of elevated cytosolic Ca^2+^ levels and Ca^2+^ homeostasis mishandling through SR Ca^2+^ leakage. Many studies using histological and expression profiling analyses reported impaired myogenesis and differentiation of cultured human DMD and mdx muscle cells and suggested that these alterations play a leading role in muscle regeneration failure [33,34,35,36,37,38,49,50]. Histological analysis of DMD muscle shows abnormal, short and small fibers with caliber variations and multiple branching. These changes are explained by the incomplete fusion of myotubes and myoblasts [51]. More recently, Farini et al. found delayed myogenesis in DMD fetal muscle, with increased PAX7 expression and downregulation of MYOD compared with healthy samples [52]. They showed that this delayed differentiation is mediated by an IP3R-dependent Ca^2+^ signaling pathway. However, in *X. laevis* skeletal myogenesis, the inhibition of the Ca^2+^/calmodulin-dependent myosin light chain kinase impairs myosin thick filament assembly, implying a potential RYR-Ca^2+^-driven mechanism [53]. In mice, blocking RYR activity with ryanodine inhibits the in vitro differentiation of fetal myoblasts [54]. In humans, *RYR1* gene mutations cause several skeletal myopathies, such as central core disease, multiminicore disease and nemaline rod myopathy [55]. *RYR1* homozygous mutant mice in which RYR1-mediated Ca^2+^ release is abolished exhibited increased apoptosis, a severely disrupted musculature with small myotubes and disarranged myofibrils [30]. Altogether, these results demonstrate that the RYR1-mediated Ca^2+^ pathway is required for skeletal myogenesis [25]. Surprisingly, Arnaudeau et al. did not find any RYR1 involvement in early human myoblast differentiation using ryanodine (1 µM). Indeed, ryanodine and caffeine release Ca^2+^ efficiently in young human myotubes, suggesting that RYR1 function is not required during the earliest stage of differentiation [56]. In healthy control myotubes, we found a decrease in myotube area and fusion index following *RYR1* silencing, suggesting a key role of *RYR1* during human myotube formation.

To our knowledge, our study is the first description of abnormal Ca^2+^ leakage from SR due to calstabin1 depletion from the RYR1 complex in human DMD skeletal muscle cells, which was only described in the mdx mouse model of DMD [38]. Gentil et al. studied neuronal nitric oxidase synthase (nNOSµ) mislocalization in patients with Becker muscular dystrophy who carry spontaneous deletions in dystrophin exons 45–55, resulting in the absence of the binding site for nNOSµ. More severe phenotypes were associated with exclusive cytosolic mislocalization of nNOSµ that correlated with RYR1 hypernitrosylation and calstabin1 release [57]. Thus, the instability of the RYR1/calstabin1 complex in dystrophinopathies is probably partly due to the absence of the dystrophin nNOS-binding domain. Furthermore, there are rising data on DMD animal models concerning the effect of RYR1 on calcium transport mishandling in mitochondria-associated membrane (MAM) contacts. In DMD, the impairment of Ca^2+^ transport between RS and mitochondria is characterized by the dysregulation of IP3R, the calcium uniport machinery and the Ca^2+^-dependent MPT pore, suggesting a complex interaction on Ca^2+^ homeostasis between these two organelles [58,59,60,61].

In the present study, administration of the RYR-calstabin1 complex stabilizing compound S107 inhibited SR Ca^2+^ leaks in DMD myotubes and increased the expression of myogenic differentiation markers. In mdx mice, stabilization of calstabin1 binding to S-nitrosylated RYR1 by treatment with S107 for two weeks reduces histological muscle damage, improves muscle function and decreases creatine kinase and calpain levels [38]. Recently, Capogrosso et al. treated mdx mice during 4 and 12 weeks with ARM210, a new Rycal compound with good oral bioavailability and distribution in skeletal muscles, and found an improvement in histologic and functional parameters, with no adverse effects [62]. Moreover, Kendall et al. reported that dantrolene, a RYR-targeting compound, which is currently used as a chronic treatment for malignant hyperthermia and muscle spasticity, synergizes with antisense-mediated exon skipping therapies in mdx mice and in inducible directly reprogrammable myotubes (iDRMs) derived from fibroblasts of patients with DMD [63]. Ryanodine and S107 also enhanced the effect of exon-skipping antisense oligonucleotides in iDRMs cells from patients with DMD [64]. The same team recently showed that dantrolene, S107 and ARM210 similarly increased antisense oligonucleotide-mediated exon skipping in DMD iDRMs and in inducible pluripotent stem cells (iPSC)-derived myotubes [63]. Furthermore, we reported that cardiac arrhythmias in mdx mice can be prevented by stabilizing cardiomyocytes RYR2 channels with S107 [65]. More recently, we proved that Rycal treatment prevents dilated cardiomyopathy development in the canine DMD model (GRMD) [66]. Altogether, these results suggest that RYR channel stabilization represents a promising therapeutic approach in patients with DMD in addition to more specific treatments such as exon-skipping therapy.

We found a correlation between elevated cytosolic Ca^2+^ concentration and endomysial fibrosis in muscle sections from patients with DMD. Desguerre et al. followed 25 patients with DMD for more than 10 years and compared 13 relevant clinical features with multiple morphometric parameters on initial muscle biopsies (myofiber size, hypercontracted fibers, necrotic-basophilic fibers, endomysial and perimysial fibrosis and fatty degeneration). They concluded that elevated endomysial fibrosis at diagnosis was the only parameter that correlated with poor motor outcome (i.e., muscle strength and age at loss of ambulation) [44]. Fibrosis is the result of reparative or reactive processes involving connective tissue, and in DMD it can be observed long before muscle degeneration [67]. Furthermore, elevated Ca^2+^ influx through calcium channels is sufficient for inducing a dystrophic phenotype in mice, including fibrosis [11]. Due to its early appearance during the disease course, a better understanding of the interactions between fibrosis development and calcium homeostasis is mandatory. It should be important to study extracellular matrix production during myogenesis in various calcium conditions, particularly in myofibroblasts that are constantly activated in DMD [67].

In summary, our results emphasize the key role of RYR1 in human DMD pathophysiology: (i) RYR1-mediated Ca^2+^ leakage through SR due to calstabin1 depletion delays skeletal muscle differentiation in DMD, and this can be improved by S107; (ii) the elevated cytosolic Ca^2+^ concentration is correlated with endomysial fibrosis, suggesting its involvement in DMD prognosis. As suggested by the promising results of combining RYR stabilizers and exon skipping therapies in DMD iDRMs, iPSC-derived myotubes [56,57] and patient iPSCs-derived cardiomyocytes [64] and in muscle function in mdx mice [38,62], our study highlights the potential effect of RYR1 stabilization as an adjunctive therapeutic strategy in patients with DMD.

## 4. Materials and Methods

### 4.1. Patients’ Samples

This study was carried out at three French neuromuscular disease centers (Montpellier, Toulouse and Saint-Etienne) from January 2012 to November 2017. Muscle biopsy samples at diagnosis were obtained from six boys (age: 4 to 8 years) with a diagnosis of DMD established on the basis of the total absence of dystrophin (no signal with the DYS1, DYS2 and DYS3 antibodies by immunochemistry and Western blotting) and the presence of dystrophin gene mutations (exon 32 duplication, exon 17 duplication, exon 56 deletion, exon 3 to 29 deletion, exon 3 to 7 duplication and exon 52 deletion). All patients were never treated at biopsy time. Three muscle samples from five healthy boys (age: 10 to 15 years) undergoing orthopedic surgery were used as controls. All boys and their parents provided a written informed consent for the scientific use of the muscle specimens, and the biopsy collection procedures and investigations on human tissue were approved by our institutional review board.

### 4.2. Histologic Diagnosis and Morphometric Analyses of Muscle Samples

All quadriceps muscle biopsies were flash frozen in isopentane pre-chilled in liquid nitrogen and stored at −80 °C. Transverse cryostat sections (10 µm) were stained with hematoxylin and eosin (HE) and Masson’s trichrome. All morphometric analyses were carried out using digitized non-overlapping consecutive images (×10, 2048 × 1536 pixels) that covered more than 80% of the tissue section surface on a Zeiss microscope (Carl Zeiss SAS—Microscopy, Marly le Roi, France). All biopsies were very well preserved, based on HE staining.

Color image segmentation of sections stained with Masson trichrome was used to assess endomysial fibrosis and muscle fiber area using the Adobe Photoshop software. Endomysial fibrosis was defined as previously described [44]. Examples of image segmentation results with pseudocolor overlay are shown in Figure 5A,B. Endomysial fibrosis and muscle fiber area were measured in 11 to 20 fascicles per biopsy.

### 4.3. Primary Human Skeletal Muscle Cell Culture

Myoblasts (muscle progenitor cells) were purified from the muscle biopsies and were cultured on collagen-coated dishes in DMEM/F12 medium with 10% fetal bovine serum (FBS), 0.1% Ultroser G and 1 ng/mL of human basic fibroblast growth factor (proliferation medium), as previously described [68]. Myoblast differentiation is detected morphologically by their fusion with each other into long multinucleated cells and by their expression of specific skeletal muscle proteins. For cell differentiation, myoblasts at 80% confluence were cultured in DMEM with 5% FBS (differentiation medium) for 3 to 4 days. The RYR-calstabin interaction stabilizer S107 (Sigma-Aldrich, St. Quentin Fallavier, France) was added to confluent cultures at 10 mM at the time of differentiation induction, and gene expression in myotubes was analyzed at day 4.

### 4.4. Transient si-RNA Transfections

The Silencer^TM^ Select *RYR1*-specific siRNA (siRYR1) and a scramble siRNA (negative control) were purchased from Fisher Scientific (Illkrich, France). Human myoblasts reaching 80% confluence were incubated with Lipofectamine RNAiMax Reagent according to the manufacturer’s recommendation (Fisher Scientific, Illkrich, France), induced to differentiate and analyzed 72 to 96 h after transfection.

### 4.5. Immunofluorescence Staining

Human myotubes were fixed in 2% paraformaldehyde (Electron Microscopy Sciences, Aigues Vives, France) in PBS and permeabilized with PBS/0.25% Triton X-100. Cells were then incubated with mouse monoclonal anti-troponin T (1/200; Sigma-Aldrich, St. Quentin Fallavier, France) antibody followed by the Alexa 555-conjugated anti-mouse secondary antibody (1/1000; Fisher Scientific, Illkrich, France). Nuclei were revealed by DAPI staining. Images (seven fields for each condition) were taken with a Zeiss epifluorescence microscope and analyzed with the ImageJ software.

### 4.6. Myotubes Area and Fusion Index Analyses

DAPI images were analyzed using the Fiji software to calculate the number of nuclei per image and normalized to the total image area to compare all conditions. A manual threshold was set to differentiate nuclei from background, and the number of nuclei was provided by the “Analyze > Analyze particles” command. Troponin T-stained myotubes were analyzed with the Fiji software (“Analyze > Measure” command) to calculate the total myotube area per image by setting a threshold to discriminate stained cells from background. Data were normalized to the total image area to compare all conditions. The fusion index was calculated as the ratio of the number of nuclei in troponin T-positive cells with at least three nuclei versus the total number of nuclei. This ratio was calculated by setting a threshold selection, inverting the selection and pasting it on the DAPI images. This approach highlights only the nuclei inside troponin T-positive cells that were then counted with the same technique used to determine the total number of nuclei.

### 4.7. Reverse Transcription Quantitative PCR

Total RNA was extracted from human skeletal muscle biopsies and primary cell cultures using the Nucleospin RNA II kit (Macherey-Nagel, Hoerdt, France). Complementary DNA (cDNA) was synthetized from 1 µg of total RNA with the Transcriptor Universal cDNA Master kit (Roche Diagnostics, Meylan, France). Then, gene expression was quantified by quantitative PCR using SYBR Green I dye chemistry on a LightCycler 480 system (Roche Diagnostics, Meylan, France). PCR primers (Supplementary Table S1) were designed using the LightCycler Probe Design software 2.0. The expression levels were determined with the LightCycler analysis software (Release 1.5.0) relative to standard curves. Data were represented as the mean level of gene expression relative to the expression of the reference gene ribosomal protein lateral stalk subunit P0 (*RPLP0*), which is suitable to normalize the mRNA expression in myoblasts cultured in differentiation conditions [69].

### 4.8. Immunoprecipitation and Immunoblot Analyses

Immunoprecipitation and immunoblot analyses of RYR1 and calstabin 1 were performed as previously described [38]. Human skeletal myoblasts were incubated in lysis buffer (35 mM NaF, 50 mM Tris maleate pH 6.8, 1 mM Na_3_VO4 and protease inhibitors). Then, 100 μg of cell lysates was incubated with a homemade anti-RYR antibody (rabbit 5029 Y2) in 0.5 mL of RIPA buffer (10 mM Tris–HCl pH 7.4, 150 mM NaCl, 5 mM NaF, 1 mM Na_3_VO4, 1% Triton-X100 and protease inhibitors) at 4 °C for 2 h, followed by protein A Sepharose beads (Sigma-Aldrich, St. Quentin Fallavier, France) at 4 °C overnight. Then, beads were washed three times with RIPA buffer, and proteins were separated on 4–20% SDS-PAGE gradient gels, blotted onto nitrocellulose membranes and incubated with rabbit 5029 Y2 anti-RYR1 (1:5000) and anti-calstabin1 (Santa Cruz Biotechnology, Heidelberg, Germany, ref.: SC-6173, 1:1000) primary antibodies at 4 °C overnight. The levels of RYR1-bound proteins were normalized to the total immunoprecipitated RYR1 (arbitrary units). All immunoblots were developed using the Odyssey system (LI-COR) and infrared-labeled secondary antibodies (Eurobio Scientific, Les Ullis, France; 1:30,000 dilution) at room temperature for 1 h. In siRNA experiments, Western blot analysis was performed using mouse monoclonal anti-RYR1 antibodies (Santa Cruz, ref.: SC-376507, 1:1000) and mouse monoclonal anti-alpha Tubulin (Sigma-Aldrich, St. Quentin Fallavier, France, T9026, 1:10.000).

### 4.9. Measurement of Intracellular Ca^2+^ Variations

Intracellular Ca^2+^ concentration variations ([Ca^2+^]_i_) were measured in cultured myotubes using the ratiometric fluorescent Ca^2+^ indicator Fura-2, as previously described [70,71]. Myotubes were cultured in Lab-Tek II^®^ chambers for 4 days, and then they were loaded with 2.5 µmol/L Fura-2AM/0.02% Pluronic F-127. After rinsing with Tyrode’s solution, chambers were mounted on a microscope stage (Axiovert, Zeiss, Iena, Germany; 20 × objective). After 15 min (the time necessary for Fura-2AM de-esterification), cells were incubated with 10 µM xestospongin C for 10 min before the addition of 100 µM 4-CMC. [Ca^2+^]_i_ variations were monitored by excitation with a dual UV light source at 340 nm and 380 nm using a lambda DG-4 excitation system (Sutter Instrument Company, Novato, CA, USA). Images were digitally captured every 0.35 s with a cooled CCD camera (Photometrics, Roper scientific, Evry, France) at 510 nm emission. Changes in [Ca^2+^]_i_ were deduced from variations in the fluorescence signal at 340 and 380 nm after correction for background and dark currents (Metafluor software, Universal Imaging Corporation, Downingtown, PA). Data were averaged, with *n* representing the number of fields.

### 4.10. Statistical Analysis

All data were expressed as the mean ± SEM unless otherwise specified. All statistical analyses were performed using the GraphPad Prism v7.0 a software. The differences between mean values were determined using the two-tailed, unpaired Student’s *t*-test with Welch’s correction. Bar graphs show the mean values ± SEM. Intracellular Ca^2+^ measurements were analyzed using a one-way ANOVA with Student–Newman–Keuls’ post hoc test. Correlations between variables were calculated using Spearman’s rank correlation coefficient (*r*). Statistical significance was determined as follows: *p*-value < 0.05 (*), *p* < 0.01 (**) or *p* < 0.001 (***).

## Data Availability

Informed consent was obtained from all subjects involved in the study.

## Supplementary Materials

The following are available online at https://www.mdpi.com/article/10.3390/ijms222312985/s1.
