# Supplementary material for: Skeletal Ryanodine Receptors Are Involved in Impaired Myogenic Differentiation in Duchenne Muscular Dystrophy Patients
Source: Int J Mol Sci. 2021 Nov 30;22(23):12985. doi: 10.3390/ijms222312985 (PMC8657486; doi:10.3390/ijms222312985)
Supplement: Supplementary file 1 [file ijms-22-12985-s001.zip › ijms-1453443-supplementary.pdf]

International Journal of
Molecular Sciences

MDPI

Supplementary Materials

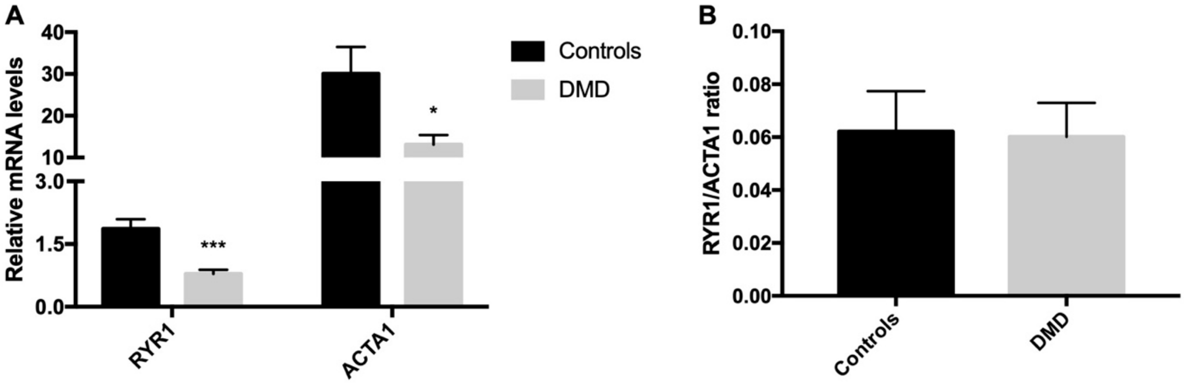

Figure S1. *RYR1* expression in total skeletal muscle extracts is similar in healthy controls and patients with DMD when normalized to *ACTA1* expression.

RYR1 and *ACTA1* relative mRNA expression levels were assessed by quantitative RT-PCR in total skeletal muscle extracts from biopsies of healthy controls (n=5) and patients with DMD (n=6). **(A)** *RYR1* and *ACTA1* mRNA expression levels were decreased in DMD samples (*RYR1*: 1.866 ± 0.228 in controls vs 0.787 ± 0.098 in DMD, p=0.0001; *ACTA1*: 30.05 ± 6.398 in controls vs 13.09 ± 2.285 in DMD, p=0.02). **(B)** The RYR1/ACTA1 ratio was similar in control and DMD samples (0.062±0.015 vs 0.060 ± 0.013, p=0.9), suggesting a similar *RYR1* mRNA expression level relative to the differentiated muscle tissue quantity. *P <0.05, ***P <0.005

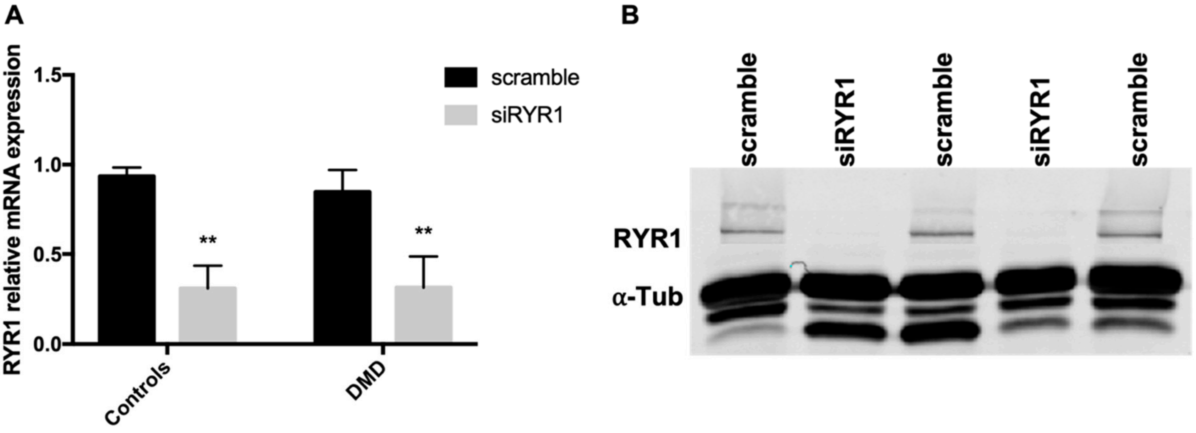

Figure S2. RYR1-specific siRNA downregulates *RYR1* mRNA expression and protein levels in human myotubes.

At 80% of confluence, myoblasts were transfected with *RYR1*-specific or scramble siRNAs and cultured in differentiation medium for 3-4 days. **(A)** *RYR1* mRNA expression levels were quantified by quantitative RT-PCR in control and DMD myotubes. **(B)** RYR1 protein levels were analyzed by western blot in control myotubes. Alpha-Tubulin protein levels (-Tub) were used as internal loading control. **P <0.01

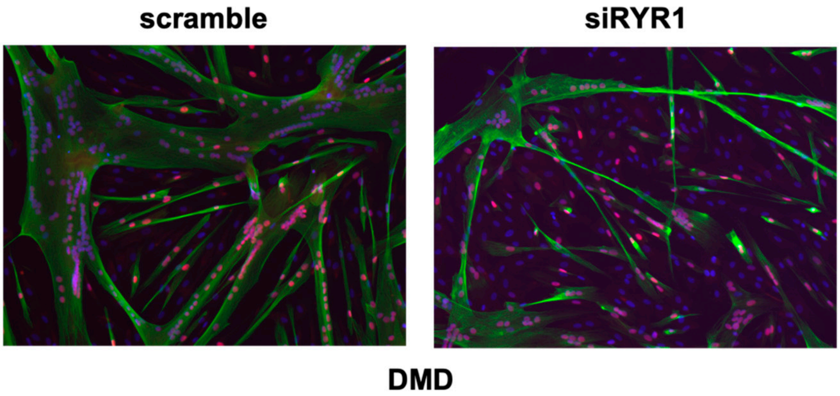

Figure S3. ***RYR1*-specific siRNA impairs myotube differentiation in DMD.**
Representative immunofluorescence staining of DMD myotubes differentiated from myoblasts transfected with RYR1-specific or scramble siRNAs. At day 3 of differentiation, myotubes were stained for troponin T (green) and DAPI (blue).

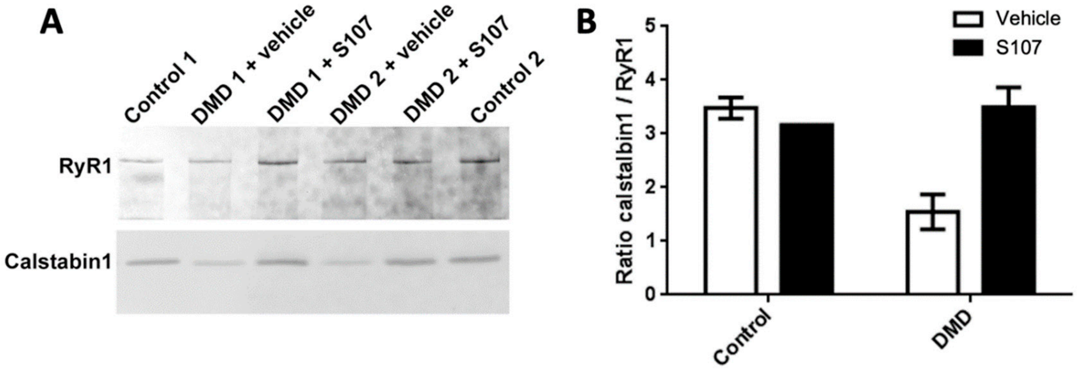

Figure S4. RYR1 depletion from calstabin1 in DMD myotubes is corrected by the RYR-RT-stabilizer S107.
(A) Representative SDS-PAGE of RYR1 and calstabin1 co-immunoprecipitation in control (n=2) and DMD (n=2) myotubes. At confluence, DMD myoblasts were incubated or not (vehicle) with S107 before switching to differentiation medium. **(B)** Quantification of RYR1/calstabin1 ratio in myotubes from healthy controls and patients with DMD incubated with vehicle (white) or S107 (black).

Table S1. Primers used for real-time quantitative RT-PCR.

Genes	Forward primer	Reverse primer	Amplicon	Accession number
Actin alpha 1 (ACTA1)	GACTTCTCAGGACGAC-GAATC	CATTTTCTTCCACAGGGCTT	187 bp	NM_001100.4
Creatine kinase, M-type (CKM)	CAAGCACCCCAAGTTCG	GTCGTCAATGGACTGGC	217 bp	NM_001824.5
Myogenic factor 5 (MYF5)	CATGCCCGAATGTAACAGTC	CCCAGGTTGCTCTGAGG	166 bp	NM_005593.3
Myogenin (MYOG)	ACCCCGCTTCTATGATGG	ACAC-CGACTTCCTCTTACACA	197 bp	NM_002479.6
Paired box 7 (PAX7)	CTGTGCCCTCAGGTTTAGT	TTCCCTTTGTCGCCCAG	151 bp	NM_002584.3
Ribosomal protein lateral stalk subunit P0 (RPLP0)	TCATCCAGCAGGTGTTCG	AGCAAGTGGGAAGGTG-TAA	224 bp	NM_001002.4
Ryanodine receptor 1 (RYR1)	AGCCGAGTCCTGGAAAG	GGCAGCAAGTTCTCAG-TAATAAG	348 bp	NM_000540.3
